# Expression regulation of *myo*-inositol 3-phosphate synthase 1 (INO1) in determination of phytic acid accumulation in rice grain

**DOI:** 10.1038/s41598-019-51485-2

**Published:** 2019-10-16

**Authors:** Ishara Perera, Ayaka Fukushima, Tatsuki Akabane, Genki Horiguchi, Saman Seneweera, Naoki Hirotsu

**Affiliations:** 1 Graduate School of Life Sciences, Toyo University, 1-1-1 Izumino, Itakura-machi, Oura-gun Gunma, 374-0193 Japan; 2 Grain Legumes and Oil Crops Research and Development Centre, Department of Agriculture, Angunakolapelessa, Sri Lanka; 3 Faculty of Life Sciences, Toyo University, 1-1-1 Izumino, Itakura-machi, Oura-gun Gunma, 374-0193 Japan; 4 National Institute of Fundamental Studies, Hantana Road, Kandy, Sri Lanka; 5 Centre for Crop Health, University of Southern Queensland, Toowoomba, QLD 4350 Australia

**Keywords:** Plant breeding, Natural variation in plants

## Abstract

Phytic acid (PA) is the primary phosphorus (P) storage compound in the seeds of cereals and legumes. Low PA crops, which are considered an effective way to improve grain nutrient availability and combat environmental issues relating to seed P have been developed using mutational and reverse genetics approaches. Here, we identify molecular mechanism regulating PA content among natural rice variants. First, we performed genome-wide association (GWA) mapping of world rice core collection (WRC) accessions to understand the genetic determinants underlying PA trait in rice. Further, a comparative study was undertaken to identify the differences in PA accumulation, protein profiles, and gene expression in low (WRC 5) and high PA (WRC 6) accessions. GWA results identified *myo*-inositol 3-phosphate synthase 1 (*INO*1) as being closely localized to a significant single nucleotide polymorphism. We found high rates of PA accumulation 10 days after flowering, and our results indicate that *INO1* expression was significantly higher in WRC 6 than in WRC 5. Seed proteome assays found that the expression of INO1 was significantly higher in WRC 6. These results suggest that not only the gene itself but regulation of *INO1* gene expression at early developmental stages is important in determining PA content in rice.

## Introduction

In cereal crops, phytic acid (PA), or *myo*-inositol 1,2,3,4,5,6-hexakisphosphate, is the primary phosphorous (P) storage molecule and accounts for 65–85% of total P in seeds^1^. Due to its strong negative polarity, PA strongly chelates with mineral cations such as potassium (K), magnesium (Mg), calcium (Ca), iron (Fe), and zinc (Zn), forming a mixed salt known as phytate. Phytates are highly insoluble salts broadly considered to be anti-nutritional compounds that prevent the absorption of important nutrients in the human intestine. Moreover, monogastric animals poorly digest PA, as they lack the enzyme phytase, which is necessary to hydrolyze P from the PA^2^. To prevent phosphorus deficiencies in animals, phosphate supplements are commonly added to diets; however, this leads to P buildup in animal waste which can result in heightened water pollution^3^. Therefore, low PA (*lpa*) crops are an effective way of mitigating these issues by increasing P bioavailability and decreasing environmental P pollution.

Several studies have been conducted with the aim of reducing PA levels in grains by developing *lpa* mutants via the impairment of PA biosynthesis or transport in rice, maize, wheat, barley, and soya bean^1,4–7^. However, the cultivation of mutant seeds containing high amounts of phosphates often results in low yields, low germination rates, and reduced seed viability^8,9^. Several rice *lpa* mutants were developed via induced mutagenesis, with the first reported mutant, KBNT *lpa1–1*, exhibiting a 45% reduction^4,10^. However, the low yields associated with mutant varieties prevent their widespread use in breeding programs. Further studies thus utilized a different strategy in attempt to reduce the phytic acid content of crops by manipulating the expression of genes related to PA biosynthesis using transgenic methods^11,12^. Transgenic rice plants have been produced which have 68% less PA content, increased Pi content, and suitable agronomic and yield traits^13^. As rice is the staple diet for more than half of the global population, improved bioavailability of micronutrients can be largely achieved through reducing its PA content.

PA biosynthesis occurs via two pathways: a lipid-dependent pathway that is common among all plant tissues, and a lipid-independent pathway which occurs predominantly in the seeds of cereals and legumes^14,15^. To date, several genes responsible for PA biosynthesis and accumulation have been identified in rice. Among these genes, 8 genes, which are expressed in developing rice grains have been identified, including *myo-inositol 3-phosphate synthase 1* (*INO1*), *inositol 1,3,4-triskisphosphate 5/6-kinase 1* (*ITPK1*), *Multidrug resistance-associated protein 13* (*MRP13*), *inositol 1, 3, 4-trisphosphate 5/6-kinase 2* (*ITPK6*), *myo-inositol kinase* (*MIK*), *inositol-pentakisphosphate 2-kinase 1* (*IPK1*), *low phytic acid 1* (*lpa1*) and *inositol 1, 3, 4-trisphosphate 5/6-kinase 2* (*ITPK2*)^16–18^. Some research reported of a slight homology of *Oslpa 1* to *2-PGK* in *Methanothermus fervidus*^19^, however it has not been functionally proven to be a *2-PGK. INO1* and *IPK1* catalyze the first and last steps of PA biosynthesis, respectively, and are considered to be major genes whose upregulation would considerably affect PA accumulation in developing seeds^16^. Phytate accumulates within protein bodies in spherical inclusions known as globoids, which are found exclusively in the aleurone layer of seeds during rice seed development^20^. However, the PA biosynthetic pathway has only recently been investigated in developing seeds^14^. Understanding the function of PA genes and the pathway and mechanisms by which they regulate PA levels in grains would facilitate the manipulation of phytic acid content in rice grains.

Genome-wide association study (GWAS) is a powerful tool which can be used to identify the complex genetic architecture of a trait by assessing the causal relationship between phenotype and genotype^21^. GWAS has been successfully used to dissect the plethora of complex traits in rice, and as a result several important associations have been identified for both quantitative and qualitative traits, including plant height, flowering time, grain yield, grain weight, amylose content, and drought tolerance^22–24^. Until now, only 2 quantitative trait loci (QTLs) related to PA content have been identified in rice, both from an ‘IR64’ x ‘Azucena’ mapping population^25^. Furthermore, proteome-wide investigations are key to understand the complex traits by the results after gene interactions and/or post-translational modifications. Recent proteomic analyses using the *lpa* rice line Os-*lpa*-XS110–1 and its parental line revealed a number of differentially-expressed proteins including stress-related proteins, storage proteins, and potential allergens^26^. Recently, we determined the PA contents of natural rice variants using GWAS^17^, but, to the best of our knowledge, this is the first report of an association mapping study using both candidate gene identification and comparative proteomics to understand functional variation among low and high PA accessions during rice grain development.

The objective of this study was to identify the physiological and genetic basis of PA accumulation in rice grains. To understand the genetic basis underlying natural variation in PA accumulation in rice, we used GWA mapping based on world rice core collection (WRC) accessions. Comparisons of gene-expression profiles and comparative proteomics analyses were carried out during the primary developmental stage, during which PA is accumulated, in both low and high PA accessions to develop the deeper understanding of the PA accumulation in rice grain.

## Results

### Variation in PA content among WRC accessions

**Figure 1:**
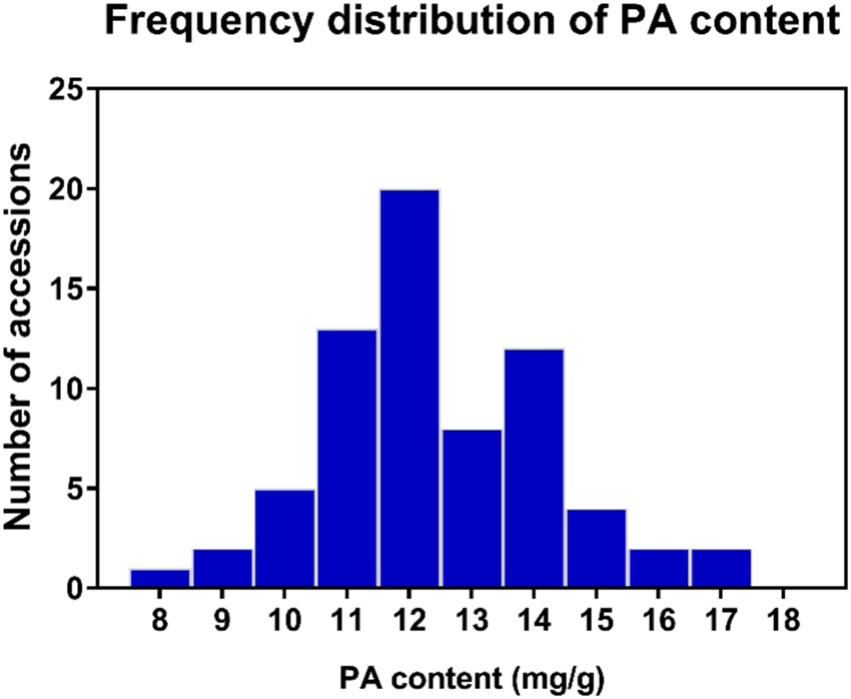
Frequency distribution of PA content in WRC accessions.

We observed high variation in PA content among WRC accessions, with PA content ranging from 8.24–17.41 mg/g (*p* < 0.05) with a mean of 12.40 mg/g (Fig. 1). The lowest and highest PA contents were recorded in WRC 5 and WRC 6, respectively^17^. Taking into consideration the *japonica, indica*, and *aus* sub-type groups, there were no significant differences among the groups in terms of PA content (*p* > 0.05).

### Genome-wide association study of PA Content

**Figure 2:**
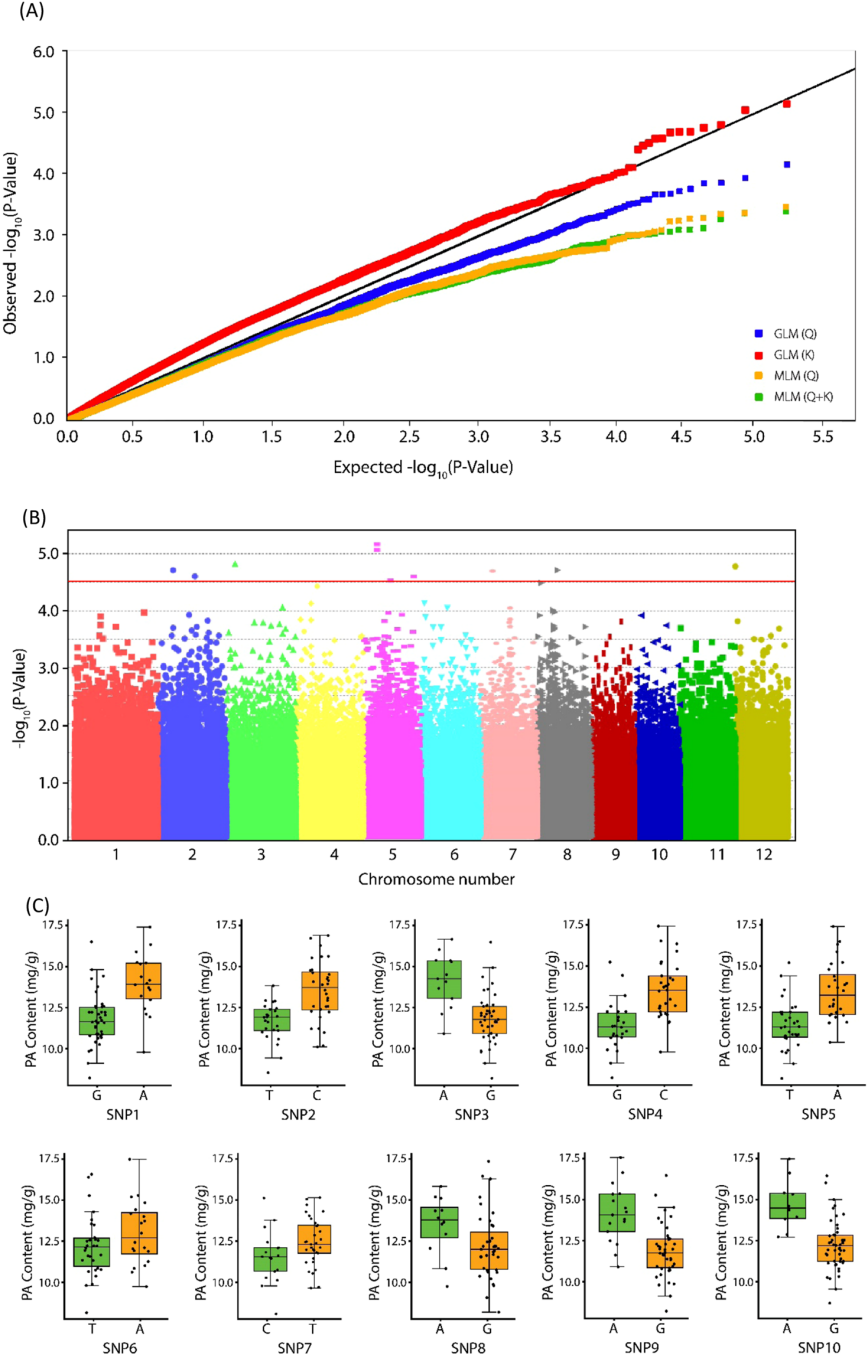
SNP-based GWAS results for PA. (**A**) Quantile-quantile (Q-Q) plot of GLM and MLM models (**B**) Manhattan plots of WRC PA content based on GWAS. Negative log10 transformed *p-*values are plotted against their position on each of the 12 chromosomes. The horizontal red line represents the genome-wide significance threshold −log_10_(P) value of 4.5 (**C**) Visualized phenotypic variation in PA content in WRC accessions for each haplotype formed by the 10 most significant SNPs (SNP 1 to SNP 10) as identified by GWAS (Table 1). Box plots were created using the ggplot2 package in R (version 3.5.3). Green and orange colors represent the minor and major alleles, respectively.

In this study, we used four models, each using different kinship matrix (K) and population structure (Q) combinations, in GWA analyses to reveal SNPs significantly associated with PA content. General Linear Model (GLM) and Mixed Linear Model (MLM) tests produced differing results and MLM Quantile-Quantile (Q-Q) plots provided clear evidence of overcorrection, which was likely caused by variation in Q and K matrices leading to a lower observed *p*-value than the expected *p*-value. In GWAS, population stratification and kinship are major confounding factors which can produce spurious results, and MLMs incorporating both K and Q matrices are generally considered more effective than GLMs and MLMs utilizing only K or Q. However, this largely depends on the genetic relationships of the association panel and phenotypic variation in the focal trait^27,28^. In our study, population structure analysis of WRC accessions showed a clustering of accessions into 3 sub-groups (Supplementary Fig. S1): *japonica*, *indica*, and *aus*. A GLM fit the data the most suitably, perhaps because no substantial phenotypic variation was present among the groups in terms of their PA content among the evaluated WRC accessions. Meanwhile GLM (K) showed the perfect distribution revealing the significance for the identification of candidate genes for PA contents of WRC accessions (Fig. 2A)

**Figure 3:**
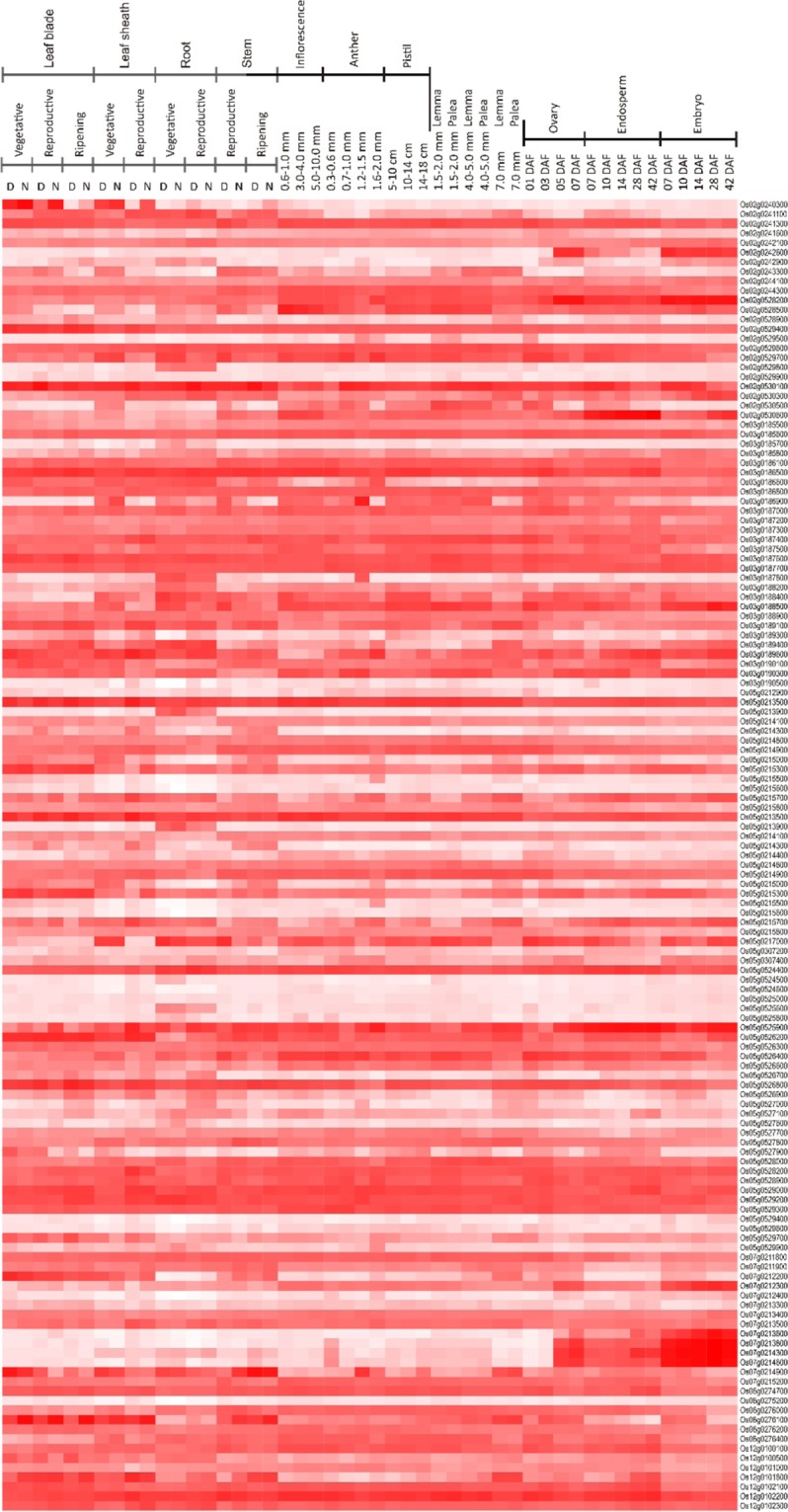
Heatmap of candidate genes identified by GWAS within 200 kbp of the most significant 10 SNPs and their expression profiles among various organs at different developmental stages in rice plants. The heatmap was created using spatio-temporal gene expression values from various tissues/organs throughout numerous developmental stages in the field^47^. The ggplot2 package in R (version 3.5.3) was used to generate the heatmap. Red indicates high expression while white indicates low expression. D: Day, N: Night, DAF; Days after flowering.

In total, 10 significant SNPs (p < 4.5 × 10^–4^) associated with PA content were detected in chromosomes 2, 3, 5, 7, 8, and 12 (Fig. 2B, Table 1). Moreover, there were significant differences in PA content among the two groups carrying the major and minor alleles among all the identified significant SNPs, except for SNP 6 and SNP 7 (Fig. 2C). Among the identified candidate genes within 100 kb upstream and downstream of peak SNPs with entries in the RAP-DB, only the genes with expression data available in the rice microarray database, RiceXPro, were used in the development of heatmaps (Fig. 3). However, no gene coding regions were co-located in association with PA biosynthesis and accumulation-related genes among the identified SNPs. SNP 3 is located on Chromosome 3 and is 226 kbp upstream of a key PA biosynthesis gene, *myo*-inositol 3-phosphate synthase 1 (*INO1*). Further, SNP 2 is located on Chromosome 2 and is 356 kbp upstream of another PA biosynthesis gene, *IPK2*. We mainly focused on developing rice grains, as PA accumulates primarily in the aleurone layer and the embryo of rice seeds throughout grain development, until 25 days after flowering (DAF)^29^. We identified 17 candidate genes with high expression (>2.0 normalized signal intensity) at both 10 and 14 DAF in rice embryos (Table 2). Among them, Os05g0525900 (zinc finger CCCH domain-containing protein 37), a transcription factor, was identified within the peak region of SNP 7 on Chromosome 5.

**Table 1:** Significant PA content-related SNPs as identified by GWAS in WRCs. MAF: Minor Allele Frequency, R^2^: Phenotypic variance explained.

SNP No.	Chromosome	Position (bp)	*p* value	Minor Allele	MAF	R^2^
SNP 1	2	8062009	1.94E-05	G	0.310	0.28
SNP 2	2	19478634	2.49E-05	T	0.439	0.27
SNP 3	3	4599691	1.5E-05	A	0.224	0.28
SNP 4	5	7081777	6.76E-06	G	0.473	0.32
SNP 5	5	7119361	8.55E-06	T	0.475	0.29
SNP 6	5	14010995	2.94E-05	T	0.440	0.30
SNP 7	5	26222539	2.52E-05	C	0.327	0.30
SNP 8	7	6223312	1.99E-05	A	0.265	0.32
SNP 9	8	10608816	1.93E-05	A	0.288	0.27
SNP 10	12	2410	1.66E-05	A	0.169	0.28

**Table 2:** Candidate genes expressed both 10 and 14 DAF (log_2_ >1.5) in the embryos of developing rice grains as identified by GWAS and gene expression heat mapping (Fig. 2).

Gene ID	Description
Os02g0242600	Similar to Glutelin.
Os02g0528200	Starch branching enzyme 3, Starch synthesis
Os02g0530100	Similar to C4-dicarboxylate transporter/malic acid transport protein, Copper chaperone homolog CCH
Os02g0530100	Copper chaperone homolog CCH.
Os03g0187700	Target SNARE coiled-coil region domain containing protein.
Os03g0188500	Glutelin family protein.
Os03g0189600	Similar to Alcohol dehydrogenase.
Os03g0190300	Similar to protein binding protein.
Os05g0213500	Rice orthologue of the abscisic acid (ABA) receptor, Positive regulator of the ABA signal transduction pathway, Abiotic stress tolerance
Os05g0525900	Similar to Zing finger transcription factor PEI1.
Os05g0529200	Crotonase, core domain containing protein.
Os07g0212300	Similar to Nudix hydrolase 16, mitochondrial precursor (EC 3.6.1.-) (AtNUDT16).
Os07g0213600	Bifunctional inhibitor/plant lipid transfer protein/seed storage domain containing protein.
Os07g0213800	Similar to Allergenic protein.
Os07g0214300	Seed allergenic protein RAG2 precursor.
Os07g0214600	Similar to Seed allergenic protein RA17 precursor.
Os12g0102200	LisH dimerisation motif domain containing protein.

### PA accumulation in developing rice grains

**Figure 4:**
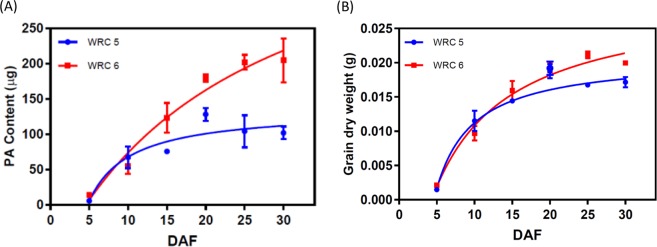
PA accumulation in developing grains of low and high PA WRCs (**A**) PA contents per grain in WRC 5 and WRC 6 (**B**) Grain dry weight of WRC 5 and WRC 6. DAF: Days after flowering.

To understand the most critical stage of PA accumulation in developing rice grains, PA content was monitored in developing rice grains from 5–30 DAF in WRC 5 and WRC 6 (Fig. 4A). At 5 DAF, PA content was very low in both accessions and had accumulated to a greater degree by 10 DAF. In WRC 5, around 66% of total PA was accumulated by 10 DAF, while in WRC 6 only around 26% was accumulated after the same amount of time. WRC 6 exhibited significantly higher PA accumulation rate than WRC 5 (p = 0.0013) at the early developmental stage (0–15 DAF). No significant differences in DW accumulation between the two accessions were observed until 20 DAF (p > 0.05), and significantly higher DW was observed in WRC 6 at 25 DAF (p < 0.05) (Fig. 4B).

### Differentially expressed proteins at 10 DAF in WRC 5 and WRC 6

**Figure 5:**
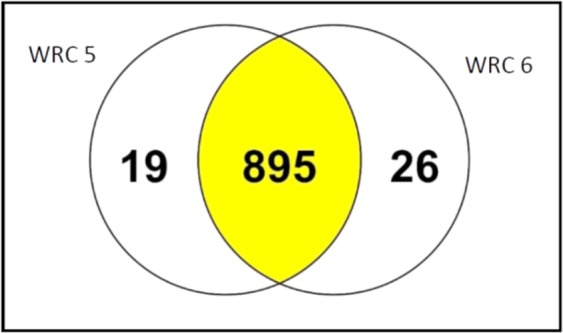
Venn diagram of the number of proteins identified by proteomic analysis for both WRC 5 and WRC 6 at 10 DAF in developing rice grains.

A total of 940 proteins were identified at 10 DAF while 895 proteins were commonly observed in both accessions at the 90% threshold level (Fig. 5A). According to their biological functions, the identified proteins were classified into several groups, with those tagged as being cellular process (31%) and metabolic process (30%) being the most abundant. Proteins were considered as being differentially expressed when they exhibited a fold-change of more than 20% and a p-value of below 0.05. Among those proteins and at this stage, 19 and 26 unique proteins were only observed in WRC 5 and WRC 6, respectively. Among PA accumulation-related proteins, *INO 1* exhibited a 1.8-fold increase (p < 0.05) in WRC 6 relative to WRC 5 (Supplementary Table S1).

### Gene sequence analysis and expression of the PA biosynthetic gene (*INO1*)

**Figure 6:**
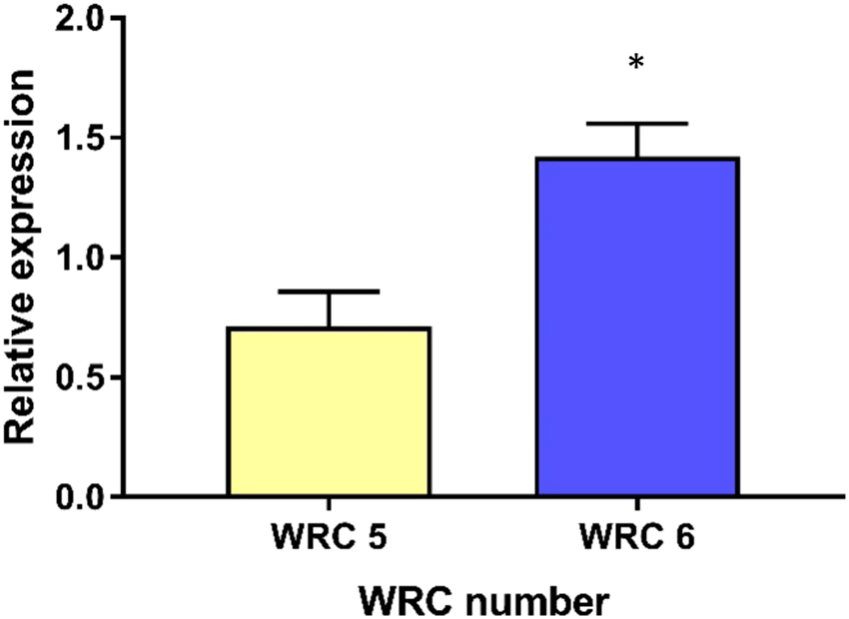
Relative gene expression of *INO1* in WRC 5 and WRC 6. Each value represents the mean ± SD of three replicates. Differences between the two accessions were evaluated using the Student’s *t*-test and significant differences between WRC 5 and WRC 6 are marked with * (*p* < 0.05).

Sequence analysis of the *INO1* gene revealed that there was no differences in coding region nucleotide sequences between WRC 5 and WRC 6 (Supplementary Fig, S2), suggesting that gene sequences have no influence over variation in PA content between the two accessions. We then evaluated the expression of the *INO1* gene at 10 DAF, when a higher PA accumulation rate was observed. The differential expression of the *INO1* gene in WRC 5 and WRC 6 resulted in a significant difference in PA accumulation between the two accessions at 10 DAF (Fig. 6). In WRC 6, which had the highest PA content, *INO1* expression was double that seen in the low PA accession. Furthermore, we did not observe any differences in the promoter region (1000 bp from the start codon) among the two accessions.

## Discussion

The WRC accessions used in this study have been developed based on a restricted fragment length polymorphism (RFLP) investigation and possess 91% of the alleles detected in the original rice collection^30^. These selected accessions were sufficiently diverse in several agro-morphological traits, and we found that the accession with the highest PA content (WRC 6) had approximately twice the PA content of the lowest recorded accession (WRC 5).

PA accumulation in WRC 5 was lower than in WRC 6 throughout seed development (Fig. 4A). Significantly higher PA accumulation rates were observed (*p* = 0.0013) at initial grain development stage (~15 DAF) in both the low and high PA accessions used in this study. Previous studies on PA accumulation have also observed rapid increases in PA content in the aleurone layer from 10 to 25 days after anthesis (DAA), with PA accumulating in the globoids from 4 DAA in both the embryo and the aleurone layer^20^. Thus, we determined 10 DAF to be the critical stage of seed development during which PA accumulation begins. We thus used this stage in our further investigations.

In this study we have done GLM and MLM tests and selected the best fit GLM (K) model to carry out GWA mapping based on the QQ plots. According to the results of GWAS, 194 candidate genes in the RAP-DB were identified within a 200-kb genomic region (Supplementary Table S2). Moreover, the significant SNPs identified by GWAS did not co-localize with known PA biosynthetic genes^31^, though *INO1* (Os03g0192700) was located close to significant SNP 3, suggesting that *INO1* plays an important role in determining PA content in rice. The PA biosynthesis pathway is initiated in developing seeds by the conversion of glucose 6-phosphate to *myo*-inositol 3- phosphate (Ins(3)P1) by *MIPS*^32^, followed by a series of phosphorylation steps. *INO1* or *RINO1*, which is a reported *MIPS* gene located on chromosome 3, is a major PA biosynthesis gene that is expressed in developing seed embryos and in the aleurone layer of rice^11,20^. On the other hand, inhibition of the first step has been suggested as the most effective way to manipulate PA biosynthesis in crops^33^. However, it has been demonstrated that knockout of *INO1* gene expression in vegetative tissues may also negatively affect plant growth and development^33^. Though we observed another PA biosynthesis related gene, *IPK2* (Os02g0523800), which is located closer to significant SNP 2, according to expression data of previous studies, *IPK2* gene showed low expression level in the developing rice grains^16,31^. Moreover, SNPs identified by GWAS did not co-localize with the previously reported QTLs for PA content^25^. The identified SNPs by this study might harbor novel genetic determinants for PA regulation. Considering these findings, we mainly focused on understanding the genetic basis of *INO1* gene in low and high PA rice accessions sourced from natural variants. We also compared the proteomes of low and high PA rice accessions at 10 DAF. Of the expressed proteins, 96% were common to both WRC 5 and WRC 6. Interestingly, among the characterized proteins, *INO1* was found to be expressed at significantly different (*p* < 0.05) levels among the low and high PA rice accessions used in this study. Similarly, the gene expression of the *INO1* gene was higher in WRC 6 grains than in WRC 5 grains at 10 DAF, which again demonstrates its importance in the PA biosynthesis pathway. Further, expression of *INO1* might be regulated by the candidate genes near significant SNPs identified by GWAS. We then sequenced the *INO1* gene to identify any causal polymorphisms which could affect PA variation among the two accessions. However, there were no differences in the gene sequences of the promoter and coding regions between the two accessions, so that the difference among these two accessions might be due to the regulation of gene expression and/or post translational modifications. This would suggest a different genetic and/or biochemical factor(s) determine the variation in *INO1* gene expression.

A major rice grain protein, glutelin, is located on chromosome 2, close to the location of SNP 2. However, no significant correlation was observed between grain PA content and glutelin content based on the analysis of 29 varieties of *japonica* rice varieties^34^. It is noteworthy that Abscisic acid (ABA) receptor (Os05g0213500) was highly expressed from 10–14 DAF in rice grains. It is likely that ABA plays a major role in seed development^35^ and, *RINO1* accumulation was upregulated by ABA treatment in cultured cells, which also suggest that ABA plays a role in PA biosynthesis and accumulation in seeds^36^. Similarly, transgenic *MIPS*-silenced rice lines exhibit lower *myo*-inositol contents correlated with altered ABA sensitiveness^12^. In Arabidopsis, mutation of the *MIPS1* gene (encoding L-*myo*-inositol 1-phosphate synthase) resulted in lower *myo*-inositol levels and increased ABA sensitivity^37^. Further, we identified the expression of Zinc finger transcription factor (Os05g0525900), which might be involved in the regulation of PA synthesis and accumulation, however no reported findings of this gene or its homology genes are related with PA accumulation. Previous studies have reported that the differential expression of transcription factor genes such as WRKY and CAMTA (Calmodulin-binding Transcription Activator) is associated with the PA biosynthesis pathway^38^. WRKY and ethylene-responsive transcription factors were also identified in a study of the low phytic acid maize line, Qi319^39^. These results suggest that the expression level of genes for PA biosynthesis like *INO1* might be regulated by complex transcription mechanisms of ABA triggered and/or WRKY, and this regulation of transcription might be the genetic basis explaining the natural variation of PA accumulation in rice.

As PA and mineral accumulation are each regulated independently^17,25,40^, it might be possible to improve the mineral bioavailability of rice grain by manipulating seed-specific *INO1* gene expression. The low PA accession, WRC 5 (as identified in this study), did not exhibit substantially lower seed weights relative to the high PA accession (Fig. 4B), and should be further investigated in future studies. Furthermore, it is evident that PA content is not mass dependent (Fig. 4). Since *INO1* regulates the early steps of the PA pathway, further investigation is required to understand the agronomic and yield performances and effective storage of other nutrients in rice grain. The results presented here confirm the importance of *INO1* expression regulation, which plays a significant role in determining PA content in rice.

## Method

### Plant materials and growth conditions

69 rice accessions of World Rice Core collection (WRC)^30^ were obtained from the National Agriculture and Food Research Organization (NARO) Genebank in Tsukuba, Japan. The grains used in this study were produced in the year 2017 at an experimental field in Itakura, Gunma, Japan or in the glasshouse at Toyo University Gunma, Japan as previously explained^17^. Based on the PA contents of these WRC accessions, three low PA accessions (WRC 5, 12, and 30) and three high PA accessions (WRC 6, 22, and 44) were selected and progressed for use in further analyses.

### Genome wide association study

#### Phenotypic data

The PA content of the 69 WRC accessions were used from previously reported^17^ measured by Phytic Acid assay kit (Megazyme International, Ireland) with minor modifications to the protocol^41^. The mean PA content based on three 3 biological replications (n = 3) for each accession were used in GWAS.

#### Association analysis

A total of 700,000 SNPs from a high-density rice array (HDRA) significant nucleotide polymorphism (SNP) database (http://www.ricediversity.org.) were used in this study^27^. Genotype data was filtered for SNPs using a minimum frequency of 0.05 for the minor allele resulting in an accession call rate of 90% and the retrieval of 47,838 filtered sites. Filtered SNPs from 62 accessions were then used to create principal components (PCs) and generate kinship matrices using centered identity-by-state methods. PCs were created by applying the principal component function using the software Trait Analysis by Association Evolution and Linkage (TASSEL) version 5.2.5^42^. Different models, including GLM with Q, GLM with K, MLM with Q, and MLM with Q + K, were created and compared to find the model which produced the best fit^43^. MLM was selected as the optimal model and analyses were run with the compression level set to the optimum and the variance component estimation set to P3D. The significance threshold was set at *p* < 4.5 × 10^–4^.

#### Candidate gene identification

The regions defined as peak SNP positions and those within 100 kbp upstream and downstream of this position were screened to identify candidate genes. All of the annotated genes within each significant SNP region were retrieved from the Rice Annotation Project Database^44^ (RAP-DB).

### Phytic acid accumulation in developing rice grains

#### Plant growth and sample collection

The selected low and high PA WRC accessions were grown in a nutrient-rich soil (Bon-sol #2, Sumitomo Chemical, Tokyo, Japan) in the glass house at Toyo University, Gunma, Japan. Seed samples from each accession were collected at 5, 10, 15, 20, 25, and 30 DAF. The samples were dried at 60 °C for 48 hours and stored in an incubator at 4 °C until their use in analyses.

#### Determination of PA content

The PA content of the rice grains at 13% moisture and at different developmental stages (5, 10, 15, 20, 25, and 30 DAF) were measured as above described. Biological samples from each developmental stage were analyzed using three replicates (n = 3).

#### Gene expression analysis

Another set of samples collected in the same manner as above were used for in gene expression analyses. The harvested seeds were dehusked and carefully stored in a refrigerator at −80 °C. Based on the results obtained from PA accumulation assays, WRC 5 and WRC 6 whole-grain samples were taken at 10 DAF, progressed, and used in gene expression analyses.

#### Designing of gene specific primers

The qRT-PCR primers and probe for the *INO1* gene (forward 5′-CAGGGTCGGGAGCTACAA-3′, reverse 5′-AAGGTCATCAGGGTTCACCA-3′ and probe #52) were designed by the assay design center (Roche Molecular systems, Inc) and GAPDH (forward 5′- GCTGCTGCTCACTTGAAGG-3′, reverse 5′-AAACATCGGAGCATCTTTGC-3′ and probe #142) was used as the reference gene. We confirmed there are no sequence differences in the priming sites of the above genes between WRC 5 and WRC 6.

#### RNA isolation, quantification and real time quantitative PCR (qRT-PCR)

Total RNA was extracted from 8–10 frozen rice grains sampled at 10 DAF. Samples from WRC 5 and WRC 6 were separately heated using a Sepasol RNAI Super G. The RNA concentration in the sample was determined using a Nanodrop 2000C spectrophotometer (Thermo Scientific). cDNA synthesis was carried out via reverse transcription reactions using the Primer script RT reagent kit according to the manufacturer’s instructions (Takara Bio Inc., Japan) using a total sample volume of 10 μL. Expression analysis was performed using real-time PCR^45^. All the experiments were performed using three biological replicates and the 2^-ΔΔCT^ method was used to calculate relative quantities^46^.

#### Proteomics

Grain samples from WRC 5 and WRC 6 taken at 10 DAF were used in proteomics analysis. The seeds were stored at −80 °C until proteome analysis was carried out using liquid chromatography-mass spectrometry (LC-MS/MS).

#### Protein extraction and nanoLC–MS/MS analysis

The grain samples were homogenized in ten volumes of lysis buffer containing 10 mM Tris–HCl pH 8.0, 7 M urea, 2 M thiourea, 5 mM (CH_3_COO)_2_Mg, 4% (w/v) CHAPS, and protease inhibitors (11697498001, Roche Diagnostics) using the Sample Grinding Kit (GE Healthcare Bio–Sciences). The homogenate was then centrifuged at 20,000 × g for 30 min at 4 °C. The supernatant was added to 100 µL reduction processing solution (100 mM NH_4_HCO_3_ containing 0.15% (w/v) DTT) and incubated at 57 °C for 30 min. After incubation, the supernatant was added to 100 µL alkylation process solution (100 mM NH_4_HCO_3_ containing 1% (w/v) IAA) and then incubated at room temperature. After adding 100 µL modified trypsin and 50 mM NH_4_HCO_3_, the mixture was incubated at 30 °C overnight. The fluid was then dried using a centrifugal concentrator (CC–105, TOMY SEIKO) and dissolved into 30 µL 0.1% formic acid. The resulting solution was then centrifuged at 20,000 × g for 10 min. The resulting supernatant was then used in nanoLC–MS/MS analyses.

Mass spectrometric analysis was carried out using nanoLC–MS/MS. The samples were loaded onto the column (75 µm internal diameter and 500 mm length: L–column2 ODS, CERI) using nano–HPLC (UltiMate 300, Dionex). The eluted peptides were analyzed by Q–Exactive Plus MS (Thermo Scientific) while the nano–LC and MS were controlled using Xcalibur (Thermo Scientific).

#### Protein identification and quantification

The nanoLC–MS/MS spectra were analyzed using the software Mascot server (version 2.5.1). The spectra data were submitted to SWISS–PROT. The following Mascot search parameters were used: threshold of the ion score cut–off, 10; peptide tolerance, 0.8 Da; MS/MS tolerance, 0.5 Da; peptide charge, trypsin as the enzyme and allowing up to two missed cleavage; carbamidomethylation on cysteines as a fixed modification, and oxidation on methionine as a variable modification.

Statistical analyses of protein spectra count data were performed using the software Scaffold (Proteosome Software, USA). Significant differences were detected using Fisher’s least significant difference test with significance set at *p* < 0.05. Only identified proteins with a false discovery rate (FDR) <0.05 were considered.

#### Plant materials, DNA extraction, and sequence analysis of the *INO1* candidate gene and its promoter region

Selected WRC 5 and WRC 6 seeds were grown in a greenhouse and DNA was extracted from the leaves 14 days after planting. Primers for sequencing the *INO1* gene and its promoter region (−1000 bp of the start codon) were designed using Primer3 software and the Nipponbare gene sequence (Supplementary Table S3) and were purchased from Thermo Fisher Scientific (Minato-ku, Japan). PCR reactions were performed and the resulting gel products were purified using the QIAquick PCR purification kit (Qiagen). DNA sequencing was performed by the Eurofins Sanger Sequencing Facility, Japan. Sequences were aligned using Multiple Sequence Alignment (https://www.ebi.ac.uk).

### Statistical Analysis

The experimental data were analyzed using the statistical analysis software Statistical Package for Social Sciences (SPSS), version 25.0. Differences between groups were analyzed using one-way analysis of variance (ANOVA) and significant differences between group means were determined using Duncan’s test at *p* < 0.05 and/or *p* < 0.001 where possible. Heat maps illustrating gene expression and associated box plots were created using the software R (version 3.5.3). Graphical representations were created using the software GraphPad Prism version 7 (GraphPad Software, San Diego, CA).

## Supplementary information

Supplementary Info

## Data Availability

The proteomics data have been deposited to the ProteomeXchange via jPOST with identifier PXD015105. DNA sequences reported here have been deposited to the GenBank database under accession of LC497076 for WRC5 and LC497077 for WRC6. The other datasets generated and/or analyzed during the current study are available from the corresponding author on reasonable request.
