# Supplementary material for: Expression regulation of myo-inositol 3-phosphate synthase 1 (INO1) in determination of phytic acid accumulation in rice grain
Source: Sci Rep. 2019 Oct 16;9:14866. doi: 10.1038/s41598-019-51485-2 (PMC6795888; doi:10.1038/s41598-019-51485-2)
Supplement: Supplementary file 1 — Supplementary Info [file 41598_2019_51485_MOESM1_ESM.pdf]

Supplementary Info

Expression regulation of *myo*-inositol 3-phosphate synthase 1 (INO1) in determination of phytic acid accumulation in rice grain

Ishara Perera[1,2], Ayaka Fukushima[1], Tatsuki Akabane[3], Genki Horiguchi[1], Saman Seneweera[4], Naoki Hirotsu[*,1,3]

[1]Graduate School of Life Sciences, Toyo University, 1-1-1 Izumino, Itakura-machi, Oura-gun, Gunma 374-0193, Japan

[2]Grain Legumes and Oil Crops Research and Development Centre, Department of Agriculture, Angunakolapelessa, Sri Lanka

[3]Faculty of Life Sciences, Toyo University, 1-1-1 Izumino, Itakura-machi, Oura-gun, Gunma 374-0193, Japan

[4]National Institute of Fundamental Studies, Hantana Road, Kandy, Sri Lanka

*****To whom correspondence should be addressed. Tel: +81 276 82 9027 Email: hirotsu@toyo.jp

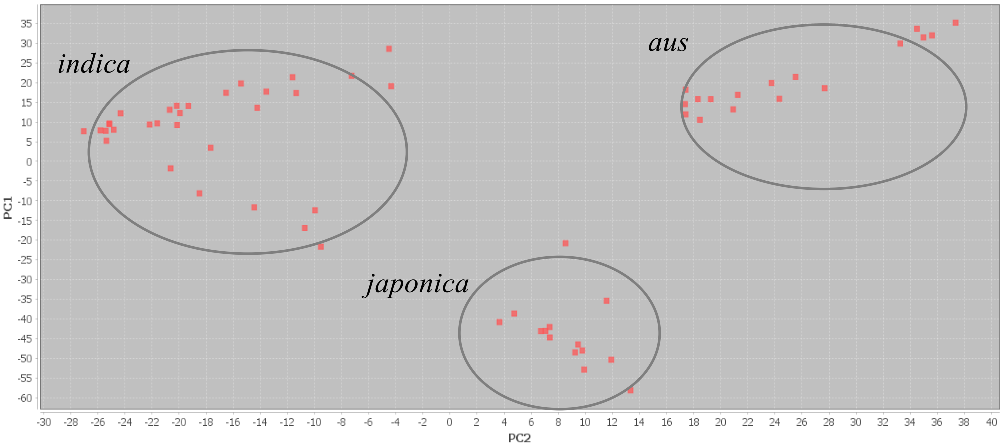

Supplementary Data Fig.S1 Clustering of WRC accessions by Principal Component Analysis in TASSEL

```
WRC5            ATTAAAAAAACCATTAATTATTTATATAGTAATCCCTCTACAATTTTTCGTGGTGATGGA
WRC6            ATTAAAAAAACCATTAATTATTTATATAGTAATCCCTCTACAATTTTTCGTGGTGATGGA
                ************************************************************

WRC5            AACATGATCACCCTGTTTGTATTTTTATCAGCAGTGATTTATCATCAGACTCTCTTTCAC
WRC6            AACATGATCACCCTGTTTGTATTTTTATCAGCAGTGATTTATCATCAGACTCTCTTTCAC
                ************************************************************

WRC5            CCTTAGGTTTGCTGAATTTCAGTGGAAAATTTGAACTCCAAACCTCTCCTGTTTAAGCTC
WRC6            CCTTAGGTTTGCTGAATTTCAGTGGAAAATTTGAACTCCAAACCTCTCCTGTTTAAGCTC
                ************************************************************

WRC5            CCATTGGTTGCCCATTTGGCCAAAATAGCTTATTTGGCTTATTAGAAAATTAAAATTAAT
WRC6            CCATTGGTTGCCCATTTGGCCAAAATAGCTTATTTGGCTTATTAGAAAATTAAAATTAAT
                ************************************************************

WRC5            TTGTAAGTAAAACTTTTATAGAACTATTCGTAGCGACTTAAAAGCCAACATTAACAAAAA
WRC6            TTGTAAGTAAAACTTTTATAGAACTATTCGTAGCGACTTAAAAGCCAACATTAACAAAAA
                ************************************************************

WRC5            AAAATTACGTTAAAAATATTTCAAAATCAAATTTAAAAATTCAAATTTTGACTTATTCTT
WRC6            AAAATTACGTTAAAAATATTTCAAAATCAAATTTAAAAATTCAAATTTTGACTTATTCTT
                ************************************************************

WRC5            TGGCTGTTTAGGCCAAACAATATAGCTATAACATTGCGACGCACGGGACGTATCAGTACT
WRC6            TGGCTGTTTAGGCCAAACAATATAGCTATAACATTGCGACGCACGGGACGTATCAGTACT
                ************************************************************

WRC5            GGTATCAAACAAGCTTTTCTAATTTTGTAACTGATCTTTTACTATAGTAGTTTCCAGCGA
WRC6            GGTATCAAACAAGCTTTTCTAATTTTGTAACTGATCTTTTACTATAGTAGTTTCCAGCGA
                ************************************************************

WRC5            TCAAATGCAAATAGCCCTCGAAAAGAAAGCAGAGACAAAACATAATAATCACTCAGAAAA
WRC6            TCAAATGCAAATAGCCCTCGAAAAGAAAGCAGAGACAAAACATAATAATCACTCAGAAAA
                ************************************************************

WRC5            CCCATCCGGAGAGGACGAACTTGCAAACACAGAACTGTATGCCTACCCACGTACTCCTAC
WRC6            CCCATCCGGAGAGGACGAACTTGCAAACACAGAACTGTATGCCTACCCACGTACTCCTAC
                ************************************************************

WRC5            GGTCCTACCTACTACCGTCAACTTGAGATTTTCTCCCTGAGAAAAACTGATTTTACCGAT
WRC6            GGTCCTACCTACTACCGTCAACTTGAGATTTTCTCCCTGAGAAAAACTGATTTTACCGAT
                ************************************************************

WRC5            TCCCCCCACGCACAACGCACGAACCTTCTCAGCTCTCACCGGTGGATAGATAGATGATAC
WRC6            TCCCCCCACGCACAACGCACGAACCTTCTCAGCTCTCACCGGTGGATAGATAGATGATAC
                ************************************************************

WRC5            TCCTCGCCTCGCCGCTTGCCTCCCCCATTGCGCTCCCACCGGCGGCCGGAGTCGTCTCCC
WRC6            TCCTCGCCTCGCCGCTTGCCTCCCCCATTGCGCTCCCACCGGCGGCCGGAGTCGTCTCCC
                ************************************************************

WRC5            AATCCGACCCAAGGTGGGGACCACCCTCCTGCTCGGTGGGCCCGAGCAGCCCGGCCAAGT
WRC6            AATCCGACCCAAGGTGGGGACCACCCTCCTGCTCGGTGGGCCCGAGCAGCCCGGCCAAGT
                ************************************************************

WRC5            GGCCGCCGAAACCCGCTATAAATCCCCCACGCTCCACCAGCCTCCGATTCCACAGCCTAC
WRC6            GGCCGCCGAAACCCGCTATAAATCCCCCACGCTCCACCAGCCTCCGATTCCACAGCCTAC
                ************************************************************
```

```
WRC5            CTAGCTTCTCCTTCTCGCTCGCTTCGCCCGCATAAGTCTAGCCACAAACCCGCCGCCGCC
WRC6            CTAGCTTCTCCTTCTCGCTCGCTTCGCCCGCATAAGTCTAGCCACAAACCCGCCGCCGCC
                ************************************************************

WRC5            GCCGCCGCCGCCGCCGCTAGCTTCCGCGGAGCGGGGCGAGCACAGCCTACCTAGCTTCTC
WRC6            GCCGCCGCCGCCGCCGCTAGCTTCCGCGGAGCGGGGCGAGCACAGCCTACCTAGCTTCTC
                ************************************************************

WRC5            CTTCTCGCTCGCTTCGCCCGCATAAGTCTAGCCACAAACCCGCCGCCGCCGCCGCCGCCG
WRC6            CTTCTCGCTCGCTTCGCCCGCATAAGTCTAGCCACAAACCCGCCGCCGCCGCCGCCGCCG
                ************************************************************

                                                 Start codon
WRC5            CCGCCGCTAGCTTCCGCGGAGCGGGGCGAGATGTTCATCGAGAGCTTCCGCGTGGAGAGC
WRC6            CCGCCGCTAGCTTCCGCGGAGCGGGGCGAGATGTTCATCGAGAGCTTCCGCGTGGAGAGC
                ************************************************************

WRC5            CCGCACGTGCGGTACGGCGCGGCGGAGATCGAGTCGGACTACCAGTACGACACGACGGAG
WRC6            CCGCACGTGCGGTACGGCGCGGCGGAGATCGAGTCGGACTACCAGTACGACACGACGGAG
                ************************************************************

WRC5            CTGGTGCACGAGAGCCACGACGGCGCCTCCCGCTGGATCGTCCGCCCCAAGTCCGTCCGC
WRC6            CTGGTGCACGAGAGCCACGACGGCGCCTCCCGCTGGATCGTCCGCCCCAAGTCCGTCCGC
                ************************************************************

WRC5            TACAACTTCCGGACCACCACCACCGTCCCCAAGCTCGGGTACGCACCCATTACCCTCGCC
WRC6            TACAACTTCCGGACCACCACCACCGTCCCCAAGCTCGGGTACGCACCCATTACCCTCGCC
                ************************************************************

WRC5            GATCGATCGATCTGTCCACCGATCGCTTTCGATCCGGCGTCCGTCCTGTTCTCGTTTTCG
WRC6            GATCGATCGATCTGTCCACCGATCGCTTTCGATCCGGCGTCCGTCCTGTTCTCGTTTTCG
                ************************************************************

WRC5            TTTTCGTCTCCCGTTTGGTCTCCGTGTTTTTCCTAGCTATAGCTAGCGCCCGATCCAGCG
WRC6            TTTTCGTCTCCCGTTTGGTCTCCGTGTTTTTCCTAGCTATAGCTAGCGCCCGATCCAGCG
                ************************************************************

WRC5            AGATGTGGGATGGCAAAAGGCCAAAGAGGGGGACTGCGAGTCTGCGGCGCCTTTTGTTGC
WRC6            AGATGTGGGATGGCAAAAGGCCAAAGAGGGGGACTGCGAGTCTGCGGCGCCTTTTGTTGC
                ************************************************************

WRC5            CTTGCTGCTCGGCCACCGTGGAAATTTGCCTGTCTCTGGGATTCGATCCCGTCTGCTCAA
WRC6            CTTGCTGCTCGGCCACCGTGGAAATTTGCCTGTCTCTGGGATTCGATCCCGTCTGCTCAA
                ************************************************************

WRC5            AACGACATTTCTTCTGAGTTTTTTTTATATCCGCTAGTTTTTATTATTATGGCTTTATCC
WRC6            AACGACATTTCTTCTGAGTTTTTTTTATATCCGCTAGTTTTTATTATTATGGCTTTATCC
                ************************************************************

WRC5            CATGGCTGCACCTGCACTGCATGTGAGGTTTCCATTAAAATTTTGCATCTGCAAGATCGA
WRC6            CATGGCTGCACCTGCACTGCATGTGAGGTTTCCATTAAAATTTTGCATCTGCAAGATCGA
                ************************************************************

WRC5            GCAGGGGGACCTACCTAGTACCATATATACCACTGGCAAGAACTCCCAAATTTATGACTG
WRC6            GCAGGGGGACCTACCTAGTACCATATATACCACTGGCAAGAACTCCCAAATTTATGACTG
                ************************************************************

WRC5            TTTATTAGCTTCTTTTATTAGTTCACCCCATTTGTTTTGTTTTTTTTTCTGTCCATGTTG
WRC6            TTTATTAGCTTCTTTTATTAGTTCACCCCATTTGTTTTGTTTTTTTTTCTGTCCATGTTG
                ************************************************************

WRC5            GCTACGTGCCAGCACACTTGTCGATCATGTTTCTTGACCCAGTTTTATGGCTGTGGGTGC
WRC6            GCTACGTGCCAGCACACTTGTCGATCATGTTTCTTGACCCAGTTTTATGGCTGTGGGTGC
```

```
                    ************************************************************

WRC5                AGGGTGATGCTCGTGGGGTGGGGCGGCAACAACGGCTCAACGCTGACGGCTGGGGTCATC
WRC6                AGGGTGATGCTCGTGGGGTGGGGCGGCAACAACGGCTCAACGCTGACGGCTGGGGTCATC
                    ************************************************************

WRC5                GCCAACAGGGAGTAAGTGATCACTCACGCTCTACTTTTCTGCCTAAGCTAGCTTCATCTG
WRC6                GCCAACAGGGAGTAAGTGATCACTCACGCTCTACTTTTCTGCCTAAGCTAGCTTCATCTG
                    ************************************************************

WRC5                TTTTTTCTTTTCTGAATTTAACTGATGATGGTGTGAGTTTAATCAGGGGAATCTCATGGG
WRC6                TTTTTTCTTTTCTGAATTTAACTGATGATGGTGTGAGTTTAATCAGGGGAATCTCATGGG
                    ************************************************************

WRC5                CGACCAAGGACAAGGTGCAGCAAGCCAACTACTATGGCTCACTCACCCAGGCGTCCACCA
WRC6                CGACCAAGGACAAGGTGCAGCAAGCCAACTACTATGGCTCACTCACCCAGGCGTCCACCA
                    ************************************************************

WRC5                TCAGGGTCGGGAGCTACAACGGGGAGGAGATCTACGCGCCCTTCAAGAGCCTCCTGCCCA
WRC6                TCAGGGTCGGGAGCTACAACGGGGAGGAGATCTACGCGCCCTTCAAGAGCCTCCTGCCCA
                    ************************************************************

WRC5                TGGTAATCTGTCCTGTTTTCTTTGACATGCTAACCACGGAAAAGATGGTAGTAGTAATAC
WRC6                TGGTAATCTGTCCTGTTTTCTTTGACATGCTAACCACGGAAAAGATGGTAGTAGTAATAC
                    ************************************************************

WRC5                ACTGCTAATGTGATAGTAGTAGTGCTAGTACAATCATAACATGGCCTTGGCCTGTTTTAA
WRC6                ACTGCTAATGTGATAGTAGTAGTGCTAGTACAATCATAACATGGCCTTGGCCTGTTTTAA
                    ************************************************************

WRC5                AGGCCAAGGTTCTAAATGAAAAGAAGAAACTTTGACTTGTGGTCATGTCCTAAAAAAGAA
WRC6                AGGCCAAGGTTCTAAATGAAAAGAAGAAACTTTGACTTGTGGTCATGTCCTAAAAAAGAA
                    ************************************************************

WRC5                AAAGATAAGTCCTGTCACAAAAGGGTATCAAAAGATAAGTCCTGTCATAAAAGGGTGTCA
WRC6                AAAGATAAGTCCTGTCACAAAAGGGTATCAAAAGATAAGTCCTGTCATAAAAGGGTGTCA
                    ************************************************************

WRC5                AAAGGTGACCATCGTGTGTCACCTTTGTCATGTTGTGATGATGCAAGTCAAAATGTTGTG
WRC6                AAAGGTGACCATCGTGTGTCACCTTTGTCATGTTGTGATGATGCAAGTCAAAATGTTGTG
                    ************************************************************

WRC5                GTACAACCACAAGGTTCCAGCATCTTTGGACAATGATTGATCTGTTGAATACATCCACAA
WRC6                GTACAACCACAAGGTTCCAGCATCTTTGGACAATGATTGATCTGTTGAATACATCCACAA
                    ************************************************************

WRC5                ATAGTTTTCAGATTTGCAGAACCTGTTTTTCCGTTTTCTGATGGTTATGAAAAAAATTCA
WRC6                ATAGTTTTCAGATTTGCAGAACCTGTTTTTCCGTTTTCTGATGGTTATGAAAAAAATTCA
                    ************************************************************

WRC5                GGTGAACCCTGATGACCTTGTGTTCGGGGGCTGGGACATTAGCAACATGAACCTGGCTGA
WRC6                GGTGAACCCTGATGACCTTGTGTTCGGGGGCTGGGACATTAGCAACATGAACCTGGCTGA
                    ************************************************************

WRC5                TGCTATGACCAGGGCAAAGGTACTTGACATTGATCTGCAGAAGCAGCTCAGACCTTACAT
WRC6                TGCTATGACCAGGGCAAAGGTACTTGACATTGATCTGCAGAAGCAGCTCAGACCTTACAT
                    ************************************************************

WRC5                GGAGTCCATGGTGCCTCTCCCCGGCATCTATGACCCCGACTTCATCGCCGCCAACCAGGG
WRC6                GGAGTCCATGGTGCCTCTCCCCGGCATCTATGACCCCGACTTCATCGCCGCCAACCAGGG
                    ************************************************************

WRC5                ATCCCGCGCGAACAATGTCATCAAGGGCACCAAGAAGGAGCAGATGGAGCAGATCATCAA
```

```
WRC6            ATCCCGCGCGAACAATGTCATCAAGGGCACCAAGAAGGAGCAGATGGAGCAGATCATCAA
                ************************************************************

WRC5            GGACATCAGGTATACTGGCATGCATGGAATTGAGCCCATTTTACTTCATAACAAAAAGGA
WRC6            GGACATCAGGTATACTGGCATGCATGGAATTGAGCCCATTTTACTTCATAACAAAAAGGA
                ************************************************************

WRC5            ATAGTATTCTGAATTAGTTATGTCTACGAGGCATGAACACTAGGGGCAGAAATAACGGAA
WRC6            ATAGTATTCTGAATTAGTTATGTCTACGAGGCATGAACACTAGGGGCAGAAATAACGGAA
                ************************************************************

WRC5            TACCTTCAGGCACCAGCTTATAACAGAAAATAGTATTCACATGATATGGTGTCAAAGCAC
WRC6            TACCTTCAGGCACCAGCTTATAACAGAAAATAGTATTCACATGATATGGTGTCAAAGCAC
                ************************************************************

WRC5            TAAATATATATTGGCACTTTCATATATCAAAAGGGCTCCCCGAAGAGTTATACTACTGAA
WRC6            TAAATATATATTGGCACTTTCATATATCAAAAGGGCTCCCCGAAGAGTTATACTACTGAA
                ************************************************************

WRC5            GTGAACTTGTTTCGTAAGATTAGTTCCAGTCTGCATTAAGATGGTTGGAACAACATGACA
WRC6            GTGAACTTGTTTCGTAAGATTAGTTCCAGTCTGCATTAAGATGGTTGGAACAACATGACA
                ************************************************************

WRC5            AACTTGTGACTATGAATGCTGTAAACATTAGACATGATATTTTTTTTATTACTTAAATAG
WRC6            AACTTGTGACTATGAATGCTGTAAACATTAGACATGATATTTTTTTTATTACTTAAATAG
                ************************************************************

WRC5            GGAGTTCAAGGAAAAGAGCAAAGTGGACAAGGTGGTGGTGTTGTGGACTGCAAACACTGA
WRC6            GGAGTTCAAGGAAAAGAGCAAAGTGGACAAGGTGGTGGTGTTGTGGACTGCAAACACTGA
                ************************************************************

WRC5            AAGGTACAGCAATGTCTGTGTTGGGCTCAATGACACAATGGAGAACCTCCTGGCGTCTGT
WRC6            AAGGTACAGCAATGTCTGTGTTGGGCTCAATGACACAATGGAGAACCTCCTGGCGTCTGT
                ************************************************************

WRC5            GGACAAGAACGAGGCGGAGATATCACCATCGACACTGTATGCCATTGCCTGCGTCATGGA
WRC6            GGACAAGAACGAGGCGGAGATATCACCATCGACACTGTATGCCATTGCCTGCGTCATGGA
                ************************************************************

WRC5            GGGTATACCGTTCATTAACGGGAGTCCTCAGAACACCTTTGTGCCTGGTATGTAATTTTC
WRC6            GGGTATACCGTTCATTAACGGGAGTCCTCAGAACACCTTTGTGCCTGGTATGTAATTTTC
                ************************************************************

WRC5            TGTGTAGAGTAGTATGCGGTTTATTTTCTCAGGTTCATGGCTTATTTGTTTACTGTGTAC
WRC6            TGTGTAGAGTAGTATGCGGTTTATTTTCTCAGGTTCATGGCTTATTTGTTTACTGTGTAC
                ************************************************************

WRC5            AGGGCTGATCGATCTTGCTATTAAGAACAACTGCCTGATTGGTGGTGATGATTTCAAGAG
WRC6            AGGGCTGATCGATCTTGCTATTAAGAACAACTGCCTGATTGGTGGTGATGATTTCAAGAG
                ************************************************************

WRC5            TGGACAGACAAAGATGAAGTCTGTCTTGGTTGATTTCCTAGTTGGTGCTGGAATAAAGGT
WRC6            TGGACAGACAAAGATGAAGTCTGTCTTGGTTGATTTCCTAGTTGGTGCTGGAATAAAGGT
                ************************************************************

WRC5            ACCAACCATATATCAAATCCTTTGGCTAGCTTTTCGCATTGAAACGTCGATATTCTTCCG
WRC6            ACCAACCATATATCAAATCCTTTGGCTAGCTTTTCGCATTGAAACGTCGATATTCTTCCG
                ************************************************************

WRC5            GAAACTTTTTTTTATATATAACCTGATAAGAACTTTGGCTTTTGTTTTCGTAGCCCACCT
WRC6            GAAACTTTTTTTTATATATAACCTGATAAGAACTTTGGCTTTTGTTTTCGTAGCCCACCT
                ************************************************************
```

```
WRC5            CAATTGTCAGCTACAACCACTTGGGGAATAATGATGGCATGAACCTTTCCGCACCTCAAA
WRC6            CAATTGTCAGCTACAACCACTTGGGGAATAATGATGGCATGAACCTTTCCGCACCTCAAA
                ************************************************************

WRC5            CATTCCGATCCAAGGAGATCTCCAAGAGCAATGTGGTCGATGACATGGTCTCAAGCAATG
WRC6            CATTCCGATCCAAGGAGATCTCCAAGAGCAATGTGGTCGATGACATGGTCTCAAGCAATG
                ************************************************************

WRC5            CCATCCTCTATGAGCTTGGCGAGCATCCTGATCATGTTGTTGTGATCAAGGTTTGCAATT
WRC6            CCATCCTCTATGAGCTTGGCGAGCATCCTGATCATGTTGTTGTGATCAAGGTTTGCAATT
                ************************************************************

WRC5            TGTCCATGGATTCTTCTGCAGCAGTATGCCATTAAAAAAAAACTTAGTTTTGGATTAAAA
WRC6            TGTCCATGGATTCTTCTGCAGCAGTATGCCATTAAAAAAAAACTTAGTTTTGGATTAAAA
                ************************************************************

WRC5            CTTGTCCATGTATTCTTCTGCAGTATGTGCCGTATGTTGGAGACAGCAAGAGGGCAATGG
WRC6            CTTGTCCATGTATTCTTCTGCAGTATGTGCCGTATGTTGGAGACAGCAAGAGGGCAATGG
                ************************************************************

WRC5            ACGAGTACACCTCAGAGATCTTCATGGGGGGTAAGAGCACCATCGTTCTGCACAACACCT
WRC6            ACGAGTACACCTCAGAGATCTTCATGGGGGGTAAGAGCACCATCGTTCTGCACAACACCT
                ************************************************************

WRC5            GTGAGGACTCACTCCTTGCCGCGCCGATCATTCTTGATCTGGTGCTCCTTGCCGAGCTCA
WRC6            GTGAGGACTCACTCCTTGCCGCGCCGATCATTCTTGATCTGGTGCTCCTTGCCGAGCTCA
                ************************************************************

WRC5            GCACCAGGATTCAGCTGAAAGCCGAGGGGGAGGTAAGAGTTCTGATGGACTGAGATCAAT
WRC6            GCACCAGGATTCAGCTGAAAGCCGAGGGGGAGGTAAGAGTTCTGATGGACTGAGATCAAT
                ************************************************************

WRC5            TGACTCTCTGCTGCTCTGCAATTGCCACCCTTTTTGCCAGCAAAATTGACTATGTTGTTG
WRC6            TGACTCTCTGCTGCTCTGCAATTGCCACCCTTTTTGCCAGCAAAATTGACTATGTTGTTG
                ************************************************************

WRC5            TTTCACTTGTTATCCTGTGCAGGAGAAGTTCCATTCCTTCCATCCAGTGGCTACCATCCT
WRC6            TTTCACTTGTTATCCTGTGCAGGAGAAGTTCCATTCCTTCCATCCAGTGGCTACCATCCT
                ************************************************************

WRC5            GAGCTACCTCACCAAGGCACCCCTTGTGAGTGCATACATGCATCTGTACTGCCCATTAAC
WRC6            GAGCTACCTCACCAAGGCACCCCTTGTGAGTGCATACATGCATCTGTACTGCCCATTAAC
                ************************************************************

WRC5            CGTTATGTTCTTTCATGATATACCAACTGATTTACTTATGTCACGAAAAATTCGTCTCCT
WRC6            CGTTATGTTCTTTCATGATATACCAACTGATTTACTTATGTCACGAAAAATTCGTCTCCT
                ************************************************************

WRC5            GGAACAGGTTCCTCCTGGCACACCAGTGGTGAACGCCCTGGCAAAGCAGAGGGCAATGCT
WRC6            GGAACAGGTTCCTCCTGGCACACCAGTGGTGAACGCCCTGGCAAAGCAGAGGGCAATGCT
                ************************************************************

WRC5            TGAGAACATCATGAGGGCCTGCGTTGGGCTGGCCCCCGAGAACAACATGATCCTGGAGTA
WRC6            TGAGAACATCATGAGGGCCTGCGTTGGGCTGGCCCCCGAGAACAACATGATCCTGGAGTA
                ************************************************************
                     Stop codon
WRC5            CAAGTGAGGGAGGCTGCCGAGGCCGAGCAGCCGTGGCATGTGGCAAAGAGGCGAATGGGA
WRC6            CAAGTGAGGGAGGCTGCCGAGGCCGAGCAGCCGTGGCATGTGGCAAAGAGGCGAATGGGA
                ************************************************************

WRC5            TGGAGCAAGAAAGACGTGATTAGCTAGCTCTAAGAATTGTTTTAGCTTCTCTTGTGATTT
WRC6            TGGAGCAAGAAAGACGTGATTAGCTAGCTCTAAGAATTGTTTTAGCTTCTCTTGTGATTT
                ************************************************************
```

```
WRC5            TTCGGTCTTTCCCATTC
WRC6            TTCGGTCTTTCCCATTC
                *****************
```

Supplementary Data Fig. S2 Comparison of the *INO1* gene and promoter sequence alignment by CLUSTAL multiple sequence alignment by MUSCLE (3.8)

Supplementary Data Table S1. Comparative abundance of proteins in WRC 5 and WRC 6 at 10 days after flowering (DAF). Fold change is presented in log2 transformation, * and ** indicates p<0.05 and **p<0.01 respectively.

**Protein Name**	**Accession Number**	**Mw (kDa)**	**Fold Change (WRC 6/ WRC 5)**	**P value**
Isoamylase 2, chloroplastic	ISOA2_ORYSJ	87	14	**
Glucose and ribitol dehydrogenase homolog	GRDH_ORYSJ	32	12	**
Aspartokinase 1, chloroplastic	AK1_ARATH	62	10	**
Receptor protein kinase-like protein ZAR1	ZAR1_ARATH	78	9.1	**
ATP synthase protein MI25	MI25_ORYSI	22	9	**
Proteasome subunit beta type-6	PSB6_TOBAC	25	9	**
Pyruvate dehydrogenase E1 component subunit alpha-2, mitochondrial	ODPA2_ORYSJ	44	9	**
Probable glutamyl endopeptidase, chloroplastic	CGEP_ORYSJ	104	8	*
Glycerol-3-phosphate acyltransferase 5	GPAT5_ARATH	56	8	*
T-complex protein 1 subunit theta	TCPQ_ARATH	59	7	*
Isoamylase 1, chloroplastic	ISOA1_ORYSJ	90	6.3	**
DNA-directed RNA polymerase subunit beta	RPOB_CHAVU	122	5.5	**
Phosphoenolpyruvate carboxylase 2	CAPP2_SORBI	110	5.2	**
Probable indole-3-acetic acid-amido synthetase GH3.4	GH34_ORYSJ	69	5	**
Phosphatidylserine decarboxylase proenzyme 3	PSD3_ARATH	70	5	*
Succinate dehydrogenase [ubiquinone] flavoprotein subunit 2, mitochondrial	SDHA2_ARATH	69	4.7	**
ATP synthase subunit beta, chloroplastic	ATPB_CAPBU	54	4.5	*
Serine/threonine-protein phosphatase PP1	PP1_ORYSJ	36	4.5	*
4-hydroxyphenylpyruvate dioxygenase	HPPD_HORVU	47	4.5	*
N-terminal acetyltransferase A complex auxiliary subunit NAA15	NAA15_ARATH	102	4.5	*
Heat shock 70 kDa protein 10, mitochondrial	HSP7J_ARATH	73	4.3	**
Kinesin-like protein KIN-14I	KN14I_ORYSJ	142	4.3	**
Plastidial pyruvate kinase 3, chloroplastic	PKP3_ARATH	63	4.2	**
Probable eukaryotic translation initiation factor 5-1	IF5Y_ARATH	49	4	**
Eukaryotic initiation factor 4A-3	IF4A3_ORYSJ	47	4	*
Seed allergenic protein RAG1	RAG1_ORYSJ	18	3.6	**
Photosystem II CP43 reaction center protein	PSBC_BRADI (+4)	52	3.5	*
Coatomer subunit beta'-1	COB21_ORYSJ	103	3.5	**
Phosphoenolpyruvate/phosphate translocator 1, chloroplastic	PPT1_ORYSJ	44	3.3	*
Xylanase inhibitor protein 2	XIP2_ORYSJ	32	3.2	**

Probable gamma-aminobutyrate transaminase 3, mitochondrial	GATP3_ORYSI (+1)	56	3.2	*
Gamma-aminobutyrate transaminase 1, mitochondrial	GATP1_ORYSI (+1)	56	3.2	*
40S ribosomal protein S15	RS15_ORYSJ	17	3	**
Actin-1	ACT1_SORBI	42	3	*
Auxin transport protein BIG	BIG_ORYSJ	552	3	**
Ubiquitin carboxyl-terminal hydrolase 13	UBP13_ARATH	131	3	*
Leghemoglobin reductase	LEGRE_VIGUN	56	2.8	*
Eukaryotic translation initiation factor isoform 4E-2	IF4E2_ORYSJ	23	2.8	*
DEAD-box ATP-dependent RNA helicase 6	RH6_ARATH	60	2.8	*
Peroxisomal acyl-coenzyme A oxidase 1	ACOX1_ARATH	74	2.7	**
Dynamin-2A	DRP2A_ARATH	99	2.4	*
Coatomer subunit gamma-1	COPG1_ORYSJ	99	2.3	*
Probable aquaporin TIP3-1	TIP31_ORYSJ	28	2.1	*
Succinate—CoA ligase [ADP-forming] subunit beta, mitochondrial	SUCB_ORYSJ	45	2.1	**
L-idonate 5-dehydrogenase	IDND_VITVI	39	2.1	*
13 kDa prolamin C	PRO25_ORYSJ	18	2	**
Pyruvate, phosphate dikinase 2	PPDK2_MAIZE	96	2	**
40S ribosomal protein S7	RS7_HORVU	22	2	*
Glyceraldehyde-3-phosphate dehydrogenase GAPC2, cytosolic	G3PC2_ARATH	37	2	*
Flavanone 3-dioxygenase 1	FL3H1_ORYSJ	42	1.9	*
DEAD-box ATP-dependent RNA helicase 15	RH15_ORYSJ (+1)	49	1.8	*
Inositol-3-phosphate synthase 1	RINO1_ORYSJ	56	1.8	*
Glutaredoxin-C6	GRXC6_ORYSJ	12	1.7	**
Maturase K	MATK_ADELA	61	1.7	**
1,4-alpha-glucan-branching enzyme 2, chloroplastic/amyloplastic	GLGB_MAIZE	91	1.6	**
Chaperonin CPN60-1, mitochondrial	CH61_CUCMA	61	1.5	*
Ubiquitin-activating enzyme E1 3	UBE13_WHEAT	116	1.5	*
Glutelin type-A 2	GLUA2_ORYSJ	56	1.4	**
40S ribosomal protein S4	RS4_ORYSJ	30	1.4	**
Peptidyl-prolyl cis-trans isomerase	CYPH_MAIZE	18	1.4	*
Mitochondrial outer membrane protein porin 1	VDAC1_ORYSJ	29	1.4	*
Cell division cycle protein 48 homolog	CDC48_SOYBN	90	1.4	*
Alpha-amylase/subtilisin inhibitor	IAAS_ORYSJ	21	1.4	*
Protein ILITYHIA	ILA_ARATH	294	1.4	*
Seed allergenic protein RA5	RA05_ORYSJ	17	1.3	**
Protein disulfide isomerase-like 1-4	PDI14_ORYSJ	62	1.3	**
Prolamin PPROL 14E	PRO7_ORYSI (+1)	17	1.2	**
Sucrose synthase 2	SUS2_ORYSJ	92	1.2	**
Cupincin	CUCIN_ORYSI	52	1.2	*
ATP synthase subunit beta, mitochondrial	ATPBM_ORYSJ	59	1.2	*
Probable glutathione S-transferase DHAR1, cytosolic	DHAR1_ORYSJ	24	1.2	*

Supplementary Data Table S2 Candidate genes identified by GWAS for PA content. Genes located within 200 kbp of the significant SNPs were obtained by Rice Genome Annotated Project Database (RAP-DB; http://rapdb.dna.affrc.go.jp/).

Gene ID	Description
Os02g0240300	Similar to Class III peroxidase GvPx2b (Fragment).
Os02g0241100	Protein kinase, core domain containing protein.
Os02g0241200	Myb-like DNA-binding domain, SHAQKYF class domain containing protein.
Os02g0241300	Conserved hypothetical protein.
Os02g0241600	Protein kinase, core domain containing protein.
Os02g0241650	Hypothetical gene.
Os02g0242100	Similar to hydroquinone glucosyltransferase.
Os02g0242550	Similar to hydroquinone glucosyltransferase.
Os02g0242600	Similar to Glutelin.
Os02g0242900	Similar to hydroquinone glucosyltransferase.
Os02g0243300	UDP-glucuronosyl/UDP-glucosyltransferase family protein.
Os02g0244000	Herpesvirus UL139, cytomegalovirus domain containing protein.
Os02g0244100	RING-type E3 ubiquitin ligase, Regulation of grain width and weight
Os02g0244300	Peptidase C19, ubiquitin carboxyl-terminal hydrolase 2 family protein.
Os02g0244450	Non-protein coding transcript.
Os02g0528200	Starch branching enzyme 3, Starch synthesis
Os02g0528300	Hypothetical gene.
Os02g0528500	Nucleic acid-binding, OB-fold domain containing protein.
Os02g0528750	Conserved hypothetical protein.
Os02g0528550	Hypothetical conserved gene.
Os02g0528900	Similar to PDR-like ABC transporter.
Os02g0529400	Plant neutral invertase family protein.
Os02g0529450	Non-protein coding transcript.
Os02g0529500	Similar to SNAP25-like protein C (Fragment).
Os02g0529600	Similar to Xyloglucan 6-xylosyltransferase (EC 2.4.2.39) (AtXT1).
Os02g0529700	Similar to Acidic ribosomal protein P2a-4 (Fragment).
Os02g0529800	Cytochrome P450 family protein.
Os02g0529850	Hypothetical protein.
Os02g0529900	Pentatricopeptide repeat domain containing protein.
Os02g0530100	Similar to C4-dicarboxylate transporter/malic acid transport protein.
Os02g0530300	Zinc finger, AN1-type domain containing protein.
Os02g0530500	Similar to ZPR3 (LITTLE ZIPPER 3); protein binding.
Os02g0530600	Similar to Poly.
Os03g0185500	Similar to binding protein.

Os03g0185600	Conserved hypothetical protein.
Os03g0185700	Transferase family protein.
Os03g0185800	Conserved hypothetical protein.
Os03g0185950	Conserved hypothetical protein.
Os03g0186100	Similar to Uroporphyrinogen III synthase.
Os03g0186500	Myoactive tetradecapeptides family protein.
Os03g0186600	Transcription factor, MADS-box domain containing protein.
Os03g0186800	Modifier of rudimentary, Modr family protein.
Os03g0186900	Radc1.
Os03g0186950	Similar to CUTA.
Os03g0187000	Similar to EMB514.
Os03g0187100	Hypothetical conserved gene.
Os03g0187200	Non-protein coding transcript.
Os03g0187300	Similar to transducin family protein / WD-40 repeat family protein.
Os03g0187350	Conserved hypothetical protein.
Os03g0187400	Zinc finger, FYVE/PHD-type domain containing protein.
Os03g0187500	Similar to Leucine Rich Repeat family protein, expressed
Os03g0187525	Hypothetical protein.
Os03g0187550	Hypothetical gene.
Os03g0187600	GRAM domain containing protein.
Os03g0187700	Target SNARE coiled-coil region domain containing protein.
Os03g0187800	Protein of unknown function DUF250 domain containing protein.
Os03g0188100	Similar to transparent testa 12 protein.
Os03g0188200	Zinc finger, RING/FYVE/PHD-type domain containing protein.
Os03g0188400	Helix-loop-helix DNA-binding domain containing protein.
Os03g0188500	Glutelin family protein.
Os03g0188900	Similar to HAHB-7 (Fragment).
Os03g0189100	Uncharacterised protein family UPF0503 domain containing protein.
Os03g0189300	Conserved hypothetical protein.
Os03g0189400	Similar to Alcohol dehydrogenase ADH.
Os03g0189600	Similar to Alcohol dehydrogenase.
Os03g0190000	VQ domain containing protein.
Os03g0190100	UbiA prenyltransferase family protein.
Os03g0190301	Non-protein coding transcript.
Os03g0190400	Hypothetical gene.
Os03g0190300	Similar to protein binding protein.
Os03g0190500	Hypothetical gene.
Os05g0212550	Hypothetical protein.
Os05g0212600	Chalcone/stilbene synthase, N-terminal domain containing protein.
Os05g0212750	Hypothetical protein.
Os05g0212900	Similar to Chalcone synthase J (EC 2.3.1.74) (Naringenin-chalcone synthase J).
Os05g0213000	Hypothetical protein.
Os05g0213150	Hypothetical protein.
Os05g0213100	Similar to Chalcone and stilbene synthases, N-terminal domain containing protein.

Os05g0213500	Rice orthologue of the abscisic acid (ABA) receptor, Positive regulator of the ABA signal transduction pathway, Abiotic stress tolerance
Os05g0213900	Virulence factor, pectin lyase fold family protein.
Os05g0214100	Similar to Kluyveromyces lactis strain NRRL Y-1140 chromosome F of strain NRRL Y- 1140 of Kluyveromyces lactis.
Os05g0214232	Hypothetical protein.
Os05g0214300	Similar to Bidirectional sugar transporter SWEET3a.
Os05g0214800	Non-protein coding transcript.
Os05g0214900	Similar to A_IG002N01.14 protein.
Os05g0215000	BURP domain containing protein.
Os05g0215066	BURP domain containing protein.
Os05g0215033	Hypothetical gene.
Os05g0215183	Conserved hypothetical protein.
Os05g0215300	UDP-glucuronosyl/UDP-glucosyltransferase family protein.
Os05g0215500	Hypothetical protein.
Os05g0215600	Protein of unknown function DUF2358 domain containing protein.
Os05g0215700	Conserved hypothetical protein.
Os05g0215800	Protein of unknown function DUF250 domain containing protein.
Os05g0216950	NB-ARC domain containing protein.
Os05g0217000	Protein of unknown function DUF1070 family protein.
Os05g0307100	Similar to tetracycline transporter protein.
Os05g0307200	Pentatricopeptide repeat domain containing protein.
Os05g0307300	Hypothetical protein.
Os05g0307400	Similar to Regulatory associated protein of mTOR (Raptor) (P150 target of rapamycin (TOR)-scaffold protein).
Os05g0309000	Hypothetical conserved gene.
Os05g0524400	Phosphofructokinase family protein.
Os05g0524525	Hypothetical protein.
Os05g0524500	Protein kinase, core domain containing protein.
Os05g0524575	Hypothetical protein.
Os05g0524600	Leucine-rich repeat domain containing protein.
Os05g0524666	Similar to Glycine-rich protein 2b.
Os05g0524732	Protein kinase, catalytic domain domain containing protein.
Os05g0524800	Malectin-like carbohydrate-binding domain domain containing protein.
Os05g0524900	Hypothetical protein.
Os05g0525000	Protein kinase, catalytic domain domain containing protein.
Os05g0525400	Malectin-like carbohydrate-binding domain domain containing protein.
Os05g0525200	Hypothetical protein.
Os05g0525501	Serine-threonine/tyrosine-protein kinase domain containing protein.
Os05g0525600	Protein kinase, catalytic domain domain containing protein.
Os05g0525701	Hypothetical protein.
Os05g0525850	Hypothetical protein.
Os05g0525800	Protein kinase, catalytic domain domain containing protein.
Os05g0525900	Similar to Zing finger transcription factor PEI1.
Os05g0526200	Similar to Calcium homeostasis regulator CHoR1.
Os05g0526300	Similar to F-box domain containing protein.

Os05g0526400	Reticulon family protein.
Os05g0526466	Non-protein coding transcript.
Os05g0526532	Non-protein coding transcript.
Os05g0526600	Zinc finger, RING/FYVE/PHD-type domain containing protein.
Os05g0526700	Harpin-induced 1 domain containing protein.
Os05g0526800	UDP-glucuronosyl/UDP-glucosyltransferase family protein.
Os05g0526900	UDP-glucuronosyl/UDP-glucosyltransferase family protein.
Os05g0527000	UDP-glucuronosyl/UDP-glucosyltransferase family protein.
Os05g0527100	UDP-glucuronosyl/UDP-glucosyltransferase family protein.
Os05g0527200	Similar to anthocyanidin 5,3-O-glucosyltransferase.
Os05g0527333	Non-protein coding transcript.
Os05g0527600	Similar to anthocyanidin 5,3-O-glucosyltransferase.
Os05g0527700	UDP-glucuronosyl/UDP-glucosyltransferase family protein.
Os05g0527800	Similar to Anthocyanidin 5,3-O-glucosyltransferase.
Os05g0527800	UDP-glucuronosyl/UDP-glucosyltransferase family protein.
Os05g0527900	UDP-glucuronosyl/UDP-glucosyltransferase family protein.
Os05g0528000	Similar to RbohAOsp (Fragment).
Os05g0528050	Hypothetical protein.
Os05g0528101	Hypothetical gene.
Os05g0528200	Similar to 50S ribosomal protein L20.
Os05g0528500	Conserved hypothetical protein.
Os05g0528600	Flavin monooxygenase-like enzyme, Auxin biosynthesis
Os05g0528701	Non-protein coding transcript.
Os05g0528900	Ribosomal protein L9 family protein.
Os05g0529000	Tonoplast-localized DUF502-containing protein, COV-like protein, Regulation of intracellular auxin transport
Os05g0528950	Non-protein coding transcript.
Os05g0529200	Crotonase, core domain containing protein.
Os05g0529300	Similar to ER lumen protein retaining receptor (HDEL receptor).
Os05g0529400	Similar to ubiquitin domain containing 1.
Os05g0529600	Hypothetical conserved gene.
Os05g0529700	Similar to electron transporter/ heat shock protein binding protein.
Os05g0529900	Similar to Cell division control protein 50.
Os07g0211800	Protein of unknown function DUF1749 family protein.
Os07g0211900	Protein of unknown function DUF632 domain containing protein.
Os07g0212200	Similar to MRNA-binding protein (Fragment).
Os07g0212300	Similar to Nudix hydrolase 16, mitochondrial precursor (EC 3.6.1.-) (AtNUDT16).
Os07g0212400	Transposase, Ptta/En/Spm, plant domain containing protein.
Os07g0213300	Pentatricopeptide repeat domain containing protein.
Os07g0213350	Non-protein coding transcript.
Os07g0213400	Conserved hypothetical protein.
Os07g0213500	Hypothetical conserved gene.
Os07g0213600	Bifunctional inhibitor/plant lipid transfer protein/seed storage domain containing protein.
Os07g0213800	Similar to Allergenic protein.
Os07g0214100	Seed allergenic protein RA17 precursor.
Os07g0214300	Seed allergenic protein RAG2 precursor.

Os07g0214600	Similar to Seed allergenic protein RA17 precursor.
Os07g0214900	Similar to Chalcone synthase C2 (EC 2.3.1.74) (Naringenin-chalcone synthase C2).
Os07g0215050	Hypothetical protein.
Os07g0215200	Conserved hypothetical protein.
Os08g0273600	Non-protein coding transcript.
Os08g0273783	Similar to Zinc finger, C2H2 type family protein, expressed.
Os08g0274150	Hypothetical genes.
Os08g0274700	Similar to TTN10.
Os08g0274775	Similar to phosphatidylinositol 3-and 4-kinase family protein.
Os08g0274700	Similar to TTN10.
Os08g0275200	Protein kinase, core domain containing protein.
Os08g0275600	Hypothetical protein.
Os08g0276000	Similar to Transmembrane 9 superfamily protein member 4.
Os08g0276100	Similar to NDF1 (NDH-DEPENDENT CYCLIC ELECTRON FLOW 1).
Os08g0276200	Similar to WRKY transcription factor 44 (WRKY DNA-binding protein 44) (TRANSPARENT TESTA GLABRA 2).
Os08g0276400	Serine/threonine protein kinase domain containing protein.
Os12g0100100	Similar to ALY protein.
Os12g0100200	Conserved hypothetical protein.
Os12g0100500	Alpha/beta hydrolase family protein.
Os12g0100700	Similar to Exo70 exocyst complex subunit family protein.
Os12g0101000	Similar to RNase P subunit p30 family protein, expressed.
Os12g0101600	WUS-type homenodomain protein, Leaf development
Os12g0101901	Hypothetical gene.
Os12g0101800	Similar to Nonphototrophic hypocotyl 1a.
Os12g0102100	Alcohol dehydrogenase superfamily, zinc-containing protein.
Os12g0102200	LisH dimerisation motif domain containing protein.
Os12g0102300	Similar to WRKY transcription factor 57.
Os12g0102350	Similar to WRKY transcription factor 57.

Supplementary Data Table S3. Primers used for *INO1* gene and promoter region sequencing

Name	Sequence
Primers used for INO1 gene sequence	
INO1-1U	TCTCCTTCTCGCTCGCTTC
INO1-1L	TTTCTTGCTCCATCCCATTC
INO1-2U	CTTCGCCCGCATAAGTCTA
INO1-2L	CCGAACACAAGGTCATCAGG
INO1-3U	AGGTGCAGCAAGCCAACTAC
INO1-3L	TATCTCCGCCTCGTTCTTGT
INO1-4U	GGACATCAGGGAGTTCAAGG
INO1-4L	AGAGGATGGCATTGCTTGAG
INO1-5U	CTGGAATAAAGCCCACCTCA
INO1-5L	GTTCTCAAGCATTGCCCTCT
INO1-6U	GGGGTAAGAGCACCATCGTT
INO1-6L	CTGCTGCTAGTTGCATAGCC
INO1-7U	CAAGTCCGTCCGCTACAACT
INO1-7L	GTAGTTGGCTTGCTGCACCT
INO1-8U	AGCTACAACGGGGAGGAGAT
INO1-8L	TGCCCTTGATGACATTGTTC
Primers used for INO1 promoter sequence	
INO_P1U	TTTTCGTGGTGATGGAAACA
INO_P1L	AGTTGTAGCGGACGGACTTG
INO_P2U	TCGTTTCAATTTCCGTTACCA
INO_P2L	CGGTGAGAGCTGAGAAGGTT
INO_P3U	ACGCACGGGACGTATCAGTA
INO_P3L	CGGAAGCTCTCGATGAACA
